# Community-driven citizen science approach to explore cardiovascular disease risk perception, and develop prevention advocacy strategies in sub-Saharan Africa: a programme protocol

**DOI:** 10.1186/s40900-020-00246-x

**Published:** 2021-02-26

**Authors:** Kufre Joseph Okop, Kathy Murphy, Estelle Victoria Lambert, Kiya Kedir, Hailemichael Getachew, Rawleigh Howe, Jean Berchmans Niyibizi, Selemani Ntawuyirushintege, Charlotte Bavuma, Stephen Rulisa, Stephen Kasenda, Effie Chipeta, Christopher Bunn, Amelia C. Crampin, Gertrude Chapotera, Abby C. King, Ann Banchoff, Sandra J. Winter, Naomi S. Levitt

**Affiliations:** 1 Chronic Disease Initiative for Africa, Department of Medicine, University of Cape Town, Cape Town, South Africa; 2 Centre for Social Science Research, University of Cape Town, Cape Town, South Africa; 3 UCT Research Centre for Health through Physical Activity, Lifestyle and Sport, Division of Exercise Science and Sports Medicine, Faculty of Health Sciences, University of Cape Town, Cape Town, South Africa; 4 Armauer Hansen Research Institute (AHRI), Addis Ababa, Ethiopia; 5 Single Project Implementation Unit, University of Rwanda, Kigali, Rwanda; 6 School of Medicine and Pharmacy, College of Medicine and Health Sciences, University of Rwanda, Kigali, Rwanda; 7 Malawi Epidemiology and Intervention Research Unit, Lilongwe, Malawi; 8 Centre for Reproductive Health, College of Medicine, University of Malawi, Blantyre, Malawi; 9 College of Social Sciences, University of Glasgow, Glasgow, UK; 10 School of Public Health and Family Medicine, College of Medicine, University of Malawi, Zomba, Malawi; 11 Department of Epidemiology and Population Health, Stanford University School of Medicine, Stanford, CA USA; 12 Stanford Prevention Research Center, Stanford University School of Medicine, Stanford, CA USA

**Keywords:** Community-driven, Citizen science, Cardiovascular disease, Risk perception, Community engagement, Participatory learning, Advocacy, Sub-Saharan Africa

## Abstract

**Background:**

In sub-Saharan Africa (SSA), which experiences a disproportionately high cardiovascular disease (CVD) burden, population-based screening and prevention measures are hampered by low levels of knowledge about CVD and associated risk factors, and inaccurate perceptions of severity of risk.

**Methods:**

This protocol describes the planned processes for implementing community-driven participatory research, using a citizen science method to explore CVD risk perceptions and to develop community-specific advocacy and prevention strategies in the rural and urban SSA settings. Multi-disciplinary research teams in four selected African countries will engage with and train community members living in rural and urban communities as citizen scientists to facilitate conceptualization, co-designing of research, data gathering, and co-creation of knowledge that can lead to a shared agenda to support collaborative participation in community-engaged science. The emphasis is on robust community engagement, using mobile technology to support data gathering, participatory learning, and co-creation of knowledge and disease prevention advocacy.

**Discussion:**

Contextual processes applied and lessons learned in specific settings will support redefining or disassembling boundaries in participatory science to foster effective implementation of sustainable prevention intervention programmes in Low- and Middle-income countries.

**Supplementary Information:**

The online version contains supplementary material available at 10.1186/s40900-020-00246-x.

## Plain English summary

Death and illnesses due to heart-related diseases is higher among people living in countries in African region compare to those in other regions of the world. Screening and prevention of heart-related diseases are usually hindered by the low levels of knowledge about these diseases and the factors causing them, and the belief that these diseases are not a threat to most people in African communities. This paper describes the processes for conducting a research that will engage members of the communities and beneficiaries as ‘citizen scientists’ to participate and lead in research initiatives. In this research, community leaders will engage trained citizen scientists who were recruited by their communities, to interview and learn how the people in their rural and urban communities consider, interpret and communicate heart-related disease threat and health risk. The country local research teams will train the citizen scientists to make use of mobile phones to gather information, and to learn together to generate knowledge and understanding to support disease prevention. The research team and citizen scientists will conduct community and stakeholders’ engagement and consultations to co-develop relevant prevention programmes for their communities. The important steps and the lessons learned in specific settings will support effective participation in research that will enable countries to identify and promote prevention programmes that are culturally suitable in low-income communities in Africa.

## Background

As the cardiovascular disease (CVD)-related death rate rises globally, sub-Saharan Africa (SSA) has a disproportionately high CVD mortality burden [1, 2]. Primary prevention of CVD targeting early identification and treatment of high-risk individuals is a proven strategy to reduce CVD burden globally [3, 4]. However, in the SSA region, effective population-based screening and prevention measures are hampered by the generally low levels of knowledge and awareness of CVD and associated risk factors, and often inaccurate perceptions of severity of risk [5].

Our previous research has demonstrated that community health workers (CHWs) in four Low- and Middle-income countries (LMICs) were able to accurately screen members of the community for CVD risk using a simple risk assessment tool, and do so more efficiently with the aid of a mobile phone app [6–8]. However, a small proportion (< 37%) of persons screened and referred for care during the study actually attended clinic for follow up in each country [9, 10]. A number of reasons for this were identified, including health system obstacles, inaccurate perceptions of severity and risk of CVD, lack of trust in CHWs to conduct CVD risk assessments, and inconsistent referral to the health system. Qualitative studies in South African low-income communities have shown that community understanding of the concept of CVD risk could be a barrier to uptake of screening [10, 11]. These findings suggest that current methods of educating communities about CVD risk, often presented to people in the form of statistics (i.e., numbers, percentages and probabilities) and communicated numerically using bar graphs, risk tables, and heart age, may not be well understood. This may lead to confusion and reduced patient actions.

In light of this situation, there are benefits to strategically involve communities at risk of CVD in developing strategies and resources to enhance their understanding and the perceived relevance of CVD risk screening [12, 13]. Exploring CVD risk perceptions, communication, and how these affect behaviours (such as participation in risk prevention activities and care-seeking) has received little attention in LMIC communities [5, 12, 14]. It has been shown that projects that take a more grounded, co-creative approach where scientists and citizens participate together in the conceptualization, data gathering, and generation of different forms of knowledge to create new understandings and a shared agenda, can increase results-oriented scientific participation and co-creation of sustainable solutions by local communities [15, 16].

**Fig. 1:**
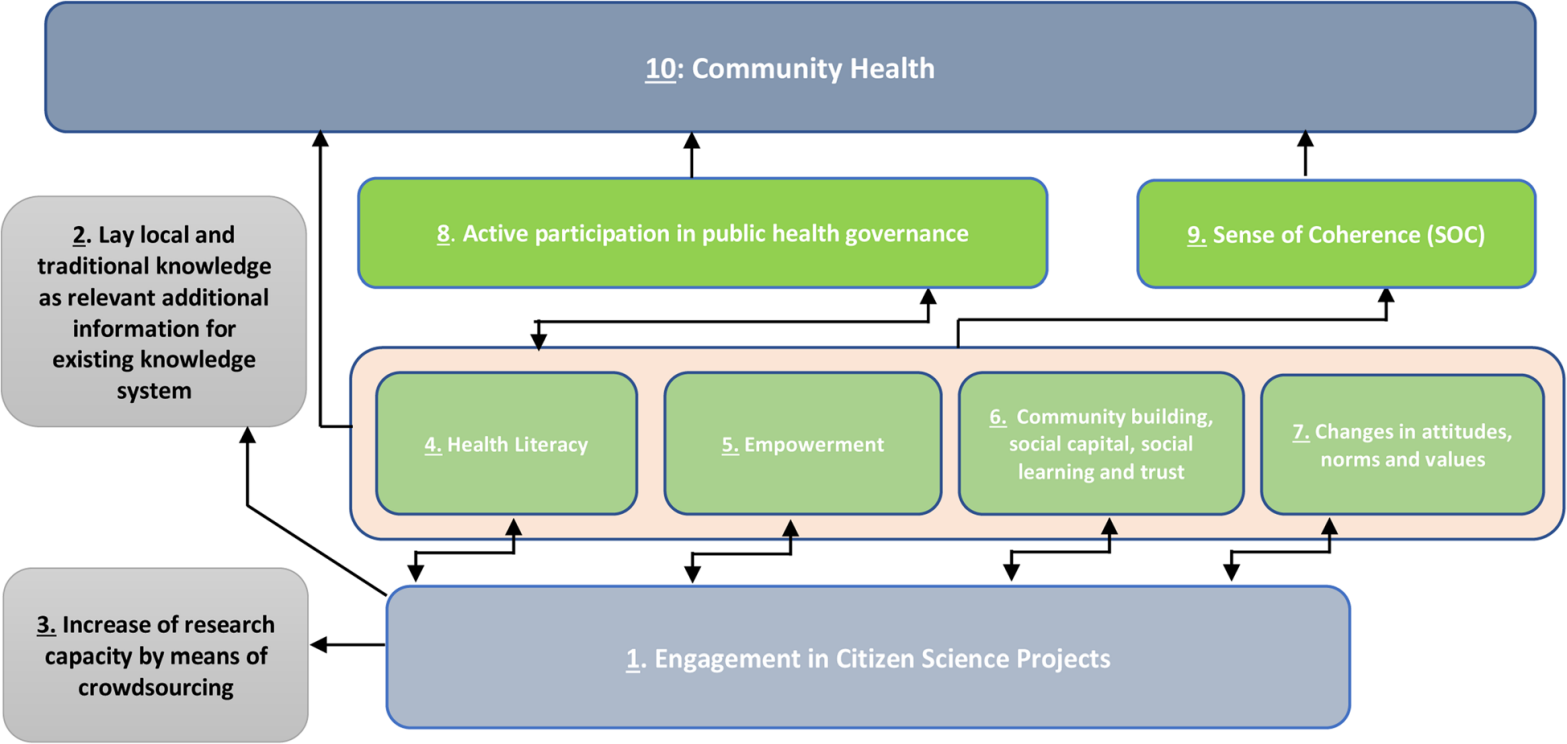
Effects of citizen science on health, health governance and knowledge system [19]. Adapted from Den Broeder et al. 2018, page 511

Citizen Science and similarly inclusive approaches like participatory action research (PAR) offer methods to increase the participation of community members in public health research. These methods have the potential to improve scientific knowledge by adding lay, local and traditional knowledge to more typical quantitative scientific methods, and can in addition, empower citizens to take social action through novel science to improve community health [17, 18] (see Fig. 1**,** below).

Citizen science originated in natural science fields, including biology, meteorology, conservation and ecology [20, 21]. It is a broad concept which often has included at least two distinguishable types: “contributory citizen science”, where citizens are approached to collect data and sometimes assist in data analysis; and “democratised citizen science” [22]. The latter type is based on the premises that science should be responsive to citizens’ concerns and needs; that the process of producing reliable knowledge can be developed and enacted by citizens themselves; and that the local, contextual and real-world knowledge of citizens can be invaluable for gaining a more ‘complete’ understanding of a phenomenon and in finding real solutions to complex problems [18, 23]. With its emphasis on robust community engagement, participatory learning, co-creation of knowledge, and advocacy for social action, this definition of citizen science is very closely aligned with participatory action research (PAR), which has been widely used in the field of public health [19, 24].

This study protocol describes the background and methods for implementing community-driven PAR, using an adaptation of the citizen science approach to explore CVD risk perceptions and develop community-specific advocacy and prevention strategies in rural and urban SSA settings. The current study is part of a larger project (Collaboration for Evidence-based Health Care and Public Health in Africa - CEBHA+) intended to develop evidence-informed policies and practices on screening approaches for hypertension, diabetes, and those at high risk of CVD in SSA communities [25].

## Methods

### Theoretical framework

The planning of this study has been based on the principles of PAR and citizen science, which overlap both in terms of philosophy and research methodology. PAR has been defined as “a philosophical approach to research that recognizes the need of persons being studied to participate in the design and conduct of all phases (e.g., design, execution, and dissemination) of any research that affects them” [26, 27]. The purpose of PAR is to foster capacity, community development, empowerment, access and social justice, and it has been widely used in public health, education, community development, agriculture and social work [27]. It is seen as a transformative process whereby researchers and participants co-create knowledge while developing a sense of community, educating each other by negotiating meanings and raising consciousness [17, 28].

Den Broeder’s (2018) descriptive framework of citizen science project characteristics provides the most recent integration of the different conceptualisations of citizen science [19].

As seen in Table 1, the first characteristic is the aim of the project; the second, the approach; and the third, the size or scope. Thus, there can be varying aims and levels of citizen engagement from A, level 1) “extreme citizen science’”, where citizens take charge of problem identification, research and knowledge production and professionals are not included to any great extent; B, level 2) “participatory science”, where citizens and researchers, NGOs and policymakers collaborate in decision-making and in co-creating relevant community-based interventions; C, level 3) “distributed intelligence”, where citizens are trained to collect, analyse and interpret data; and D, level 4) “crowdsourcing”. Here, through participatory method information or input on a particular issue or project are obtained by enlisting the services of a large number of people, typically through web/internet.

**Table 1:** Citizen Science descriptive characteristics (Adapted from Den Broeder et al. 2018, page 507)

**AIMS**	1. Investigation: aimed at answering scientific questions
	2. Education: aimed at educational goals
	3. Collective goods: public health, management of infectious disease, protect and manage natural resources
	4. Action: citizens and scientists collaborate to address local concerns through advocacy and community engagement
**APPROACHES**	A: Extreme (absolute) Citizen Science: Citizens in charge from problem definition, data collection and analysis, to interpretation and knowledge development
	B: Participatory Science: Participation of citizens in problem definition and data collection
	C: Distributed Intelligence a. Citizens as basic interpreters b. Volunteered thinking
	D: Crowd Sourcing a. Citizen as sensors b. Volunteered computing c. Group-based reasoning and advocacy
**SIZE**	1. Local 2. Mass

### Study design and study population

**Fig. 2:**
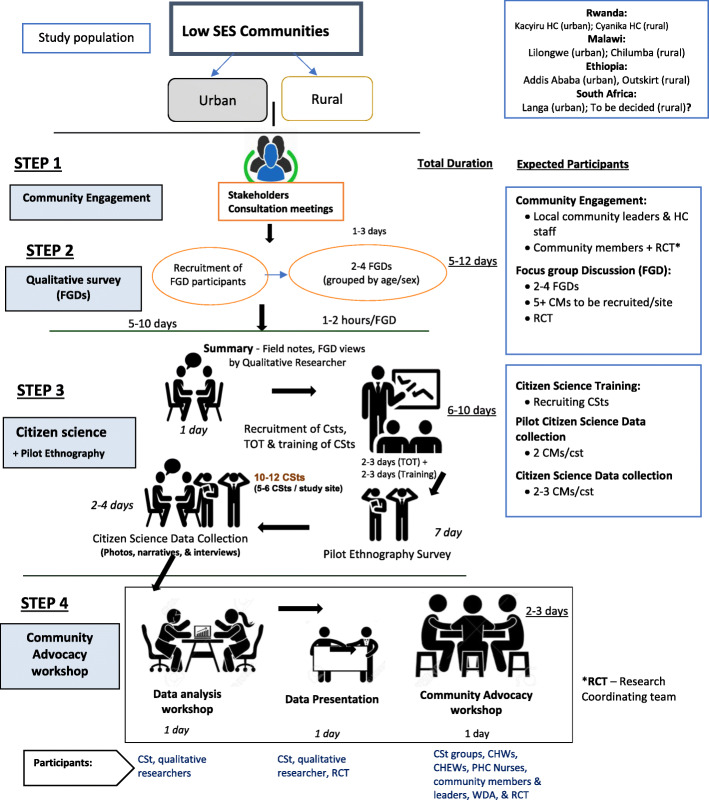
Project design and activity workflow

The primary aim of the current study is to describe the background and methods for implementing community-driven PAR, using an adaptation of the citizen science approach to explore CVD risk perceptions and develop community-specific advocacy and prevention strategies in rural and urban SSA settings. The study reflects participatory action research using citizen science processes. The research project design and work flow are presented in Fig. 2. These involve a systematic process that will begin with community engagement and consultation meetings, then fact-finding survey, and focus group discussions, and will end with citizen science processes and advocacy.

The study will be conducted in four African countries– Rwanda, Malawi, Ethiopia and South Africa. Two communities (rural and urban) are being purposively selected in each country, making a total of eight communities). The study communities are considered as catchment areas of a designated health centre with which CEBHA+ will partner for the planned population-based CVD risk screening and care using CHWs. The selection of the communities is dependent on the decisions reached by the community stakeholders during their community engagement consultations, and this decision will also consider the prevalence of non-communicable diseases (NCDs) in the study communities. The target population consists of men and women from lower socio-economic status (SES) communities, aged 18–65 years. The age limit was agreed upon by the project team, basically considering economically productive years in African setting.

#### Sampling of study participants

In each country, 8–12 villages are being purposively selected from the rural and urban study communities. A minimum of 12 community members (at least six from each study site) will be recruited and trained as citizen scientists from the two communities within each country. Recruitment will be done with the help of community stakeholders (which will include local community organizations, leaders, and/or relevant non-governmental organizations) during a community consultation meetings in the selected countries.

### Capacities of the research teams

Two of the investigators (KO, EL) are members of the *Our Voice* Global Network for Citizen Science and Health Equity, developed by researchers and community engagement experts from Stanford University (ACK, AB, SJW), and have been involved in the design and implementation of Citizen Science projects and evaluation.

NL had led the researchers in the Chronic Disease Initiative for Africa (CDIA) of University of Cape Town to implement CVD risk screening assessments in communities of four LMICs, namely, South Africa, Guatemala, Bangladesh, and Mexico [7, 9]. KO and three researchers from CDIA have conducted further trainings with members of the research teams in the countries during regular virtual meetings and in-person visits.

### Engagement and capacity development for country research teams

Initial engagement with the project teams has been undertaken in three of the countries (Rwanda, Malawi, and Ethiopia) following the approval of the research by the respective country ethics committees. Engagement took the forms of discussions and collaborative networking during annual CEBHA+ Research Network meetings that have taken place in Tanzania in 2017, Uganda in 2018, and Malawi in 2019 – following brief stakeholders mapping. To further enhance the skills for implementation and coordination of the study in the respective countries, capacity development of the country research teams will be undertaken using on-site training visits, and virtual and engagement interactions through Skype, Zoom, Microsoft Teams, telephone calls, etc. KO also visited each participating country to conduct onsite training of project teams on designing country-adapted citizen science approaches, including co-designing specific data collection tools, the mobile data collection system, and data analysis, presentation and advocacy.

The research team from CDIA will be providing further capacity development and harmonization of study processes and procedures remotely and in-person (where possible). The capacity development will include live role-plays enabling researchers, research assistants and coordinators to facilitate focus group discussions (FGDs) and stakeholder and citizen scientist advocacy training. The meetings will also be an avenue to practice data entry and provide supervision on the use of the EpiCollect mobile data collection system.

Face to face meetings with citizen scientists groups [6–15] and country project teams will be undertaken. For this, COVID-19 prevention procedures will be adhered to as stipulated in each country or region. The project PI in each country will ensure that citizen scientists and the project team adheres to COVID-19 prevention procedures (e.g., wearing of face-mask, regular hand-sanitization, and social distancing). Face-mask and had sanitizers will be provided as needed.

The following training activities will be undertaken to develop the capacities of the research teams and the trained citizen scientists:

- The *facilitation and analysis of qualitative data:* a 2-day virtual workshop for country research teams to support facilitation of FGD, analysis and reporting of qualitative research data.
- *Citizen Science and data collection:* a 3-day Train-The-Trainers workshop for project teams on Citizen Science and mobile (EpiCollect) data collection. This will be followed by a 2-day pilot-testing of mobile data collection too (EpiCollect App); and a 5-day walk-along Citizen Science interview (i.e. actual data collection).
- *Stakeholders’ Advocacy workshop:* 1-day training to enhance the skills of the trained citizen scientists on data presentation and engagement with stakeholders. This will be followed by a 1-day community advocacy workshop (in each study community) to present the findings from citizen science and discuss the implications with the focus on determining the possible strategies and steps to communicate risk in a culturally appropriate and effective way.

### Community and stakeholders’ engagement

Community and stakeholder engagement is planned as an initial step of this proposed study, and this is to facilitate proper community entry, consultations and engagement with relevant stakeholders in the project communities and regions in the project countries. The community engagement process will be facilitated by the research coordinating team in the respective countries. This will involve community visits, stakeholders’ review, consultation meetings, and discussion of the focus of the research enquiry, possible study methods and procedures, including what adaptations might need to be made in each region. The country research teams will first undertake a stakeholder mapping and identification, and will meet with key community leaders to share the project goals. This will be followed by further identification of relevant stakeholders in the community (e.g., community leaders and organizations, and CHWs-supporting organizations, health institutions, local health committee teams, and women organizations). Subsequently, 2–5 community meetings and consultations with the community stakeholders will be held in each project’s two sites (i.e., rural, urban). This process will start with meetings of project teams to clearly defined research objectives, scope, and what the study is intended to achieve – that is, undertaking community-led citizen science to support risk perception learning, communication and advocacy by community members for CVD prevention. The focus of the engagement will be co-learning, co-designing, and co-creation of learning and solutions.

### Qualitative enquiry (community-wide data collection)

We will undertake community-wide qualitative data collection to obtain first-hand contextual insights on each community’s perceptions of CVD, and learn about risk perception, interpretation, and presentation options in the different settings. We will accomplish this through rapid ethnographic (fact-finding) surveys and semi-structured focus group discussions, which is being used to inform subsequent development of the citizen science engagement mobile data collection tool, described below. This qualitative enquiry will facilitated by the respective countries project teams with support from the identified community stakeholders.

#### Rapid ethnographic (fact-finding) survey

Prior to the focus group discussions and the citizen science interviews, eligible community members will be recruited and trained as study citizen scientists by the local researchers to conduct a brief ‘fact finding’ pilot ethnographic survey. This will involve each citizen scientist interviewing two community members using mobile phones to collect photovoice and narratives. Community members will be recruited by the study team, and the citizen scientists. The community members will be selected from the respective communities where the citizen scientists resides, using sampling method convenient to each country (either purpose or random sampling). Random sampling method will involve selecting every second compound in the communities beginning from a central point in the study communities. The purpose of this phase is to provide training, practice and support for citizen science data collection, with the citizen scientists learning to gather preliminary information, in a structured format, on what people know about CVD and the associated risk factors. The information gathered as well as those from FGDs will be used to develop a citizen science interview guide and questions for the mobile app (EpiCollect).

#### Focus group discussions (FGDs)

Focus groups are intended to elicit information on knowledge and perceptions of CVD risk in the community, and will be led by a team of two qualitative researchers and the project staff members. With the use of the volunteer “snowball” sampling strategy [29], community members will be recruited within the catchment area of the designated health centres. They will be selected based on individual country administrative structure and public health system (see example of Ethiopia administrative and public health system overview in Appendix 1). In addition, we will seek to utilize the existing forums for social cohesion at the village level, such as women and men organizations, health committees in the villages and districts, and community health extension programmes structures. In order to address our study objectives, the study sampling process will take into consideration the socioeconomic status (SES) (i.e., rural and urban location, education) of the targeted population. To maintain homogeneity amongst FGDs participants, four FGDs will be conducted in each study site (rural/urban). Two FGDs will be conducted with men and women (young and older groups) separately, and will consists of 6–12 participants. The focus groups will be undertaken with adults of the same age categories (18–65 years) for a maximum duration of 2 h. We will aim to recruit approximately men and women in separate groups to participate in the focus groups. Pictographs, risk communication cues cards, numeric values cards (i.e., risk score ranges in thermometer form, and pictures), risk score charts and relevant audio-visual materials will be used to facilitate the group discussions. Focus groups will be audio-recorded with permission from the group members; and consent for this has been secured in the countries. The proceedings in each group session will also be documented by note-taker. FGDs will be translated and transcribed into English for data analyses.

### Citizen science

#### Process for recruiting citizen scientists

Citizen scientists will be recruited from within each community to interview fellow community members Depending on the country, during the community meetings, local community stakeholders and opinion leaders (e.g., chairs of Council of Health Extension workers (HEWs), and Women Development Army in Ethiopia; Health Advisory Committees in Malawi; and Local Health Committees in Rwanda) will be requested to identify and agree on one opinion leader who is suitable person to be recruited as citizen scientists in each selected village. Criteria for selection of community members as citizen scientists will be finalized by the respective communities. The inclusion criteria will be men and women aged 18 to 65 years who lives in the project community for more than 2 years. The following attributes have been suggested by the project teams as preferred qualities of a citizen scientist: personal advocacy skills, confidence to take action to improve one’s life, participation in building and maintaining community, having a strong social bond and mutual trust in the community and the ability to engage in the recommended CVD behaviours, including, not currently smoking, physically active, and or advocate for healthy lifestyle [30].

### Incentives for citizen scientists

The citizen scientists are community volunteers who will be identified and selected by the communities to support participatory research, learning and advocacy for a healthy community. They will be provided with incentives to support their transport and refreshment during the active period of their engagement in the project implementation. The incentives may include vouchers or monetary equivalent. To ensure participation and sustainment, the value of the incentives will be decided by the respective communities during stakeholders’ engagement meetings. In addition, citizen scientists will receive training on CVD and NCDs prevention, and use of mobile data collection tool (EpiCollect). Each of the citizen scientist will be provided with a mobile device (i.e., tablet, or mobile phone or Ipad) to support data collection. These devices will be returned to the project team supervisor after each day’s data collection.

#### Collaborative and co-designing of EpiCollect questions & mobile data collection tool

The research teams in the respective countries are engaged with the citizen scientists to co-design the questionnaires that will be used to support mobile data collection using a free and open source data collection application, the EpiCollect. The questionnaire to be used for mobile data collection using the EpiCollect app will be developed based on the findings from the rapid ethnography and FGD findings. In addition, the questionnaire will be pilot-tested by members of the target population, and then revised. This will then be translated into dialects of the selected six communities. The EpiCollect app will be used to collect photos and accompanying audio on CVD risk perception, and frequently used communication channels in the communities. Pilot-testing of the EpiCollect tool, data collection, extraction, and analysis will be facilitated by each country’s research team with support from the CDIA team.

#### Sampling of participants for citizen scientist interviewing

Trained citizen scientists in each country will be guided to go out to their communities and collect data from 4 to 5 eligible community members with mobile phones using the EpiCollect app during walk-along interviews [24]. Each of the six citizen scientists selected will recruit at least 4 community members to whom they will administer a walk-along semi-structured interview. Characteristics considered for recruitment include age and gender, level of education, and CVD risk factors. The community members selected for interview should have known risk factor(s). Risk factors considered include reported tobacco use or smoking, high alcohol intake, physical inactivity, and/or morbidity reported (i.e. diagnosed with diabetes, hypertension, or previous stroke, angina, myocardial infarction, etc.), and certain self-reported psychosocial factors (stress, anxiety, depression).

### Citizen science mobile data collection

#### EpiCollect data collection tool

Citizen Scientist will collect data during the citizen science walk-along interviews using *EpiCollect* Data collection mobile app deployed to mobile devices (cell phone, iPad or tablet) for use in data collection.

The questionnaire comprises participants demographics (country, location, age, and gender), and sets of questions and accompanying photos on general health risk, perceived CVD risk, communication of risk, and health seeking, The data to be collected will include 5–6 photos and narratives. Data will be collected by trained citizen scientists using the guides in Table 2 below. Prior to the training of the citizen scientists, each country research team co-designed the interview tools (EpiCollect Questionnaire) with the citizen scientists.

**Table 2:** Processes used by citizen scientists to collect data

**Role of Citizen Scientist during data collection process will be as follows.**	
The Citizen Scientist will:	
Identify a potential participant and explain study based on study protocol	
• consent a willing participant	
• entered the participants details on his mobile app (EpiCollect) data collection tool)	
• guide consented participant on data collection with EpiCollect mobile app	
• guide the participant to capture pictures and audio records by themselves.	
• Upload data (photos and narratives) online	
The project staff oversee the Citizen Scientists and support their choices of participants to ensure that they meet study eligibility criteria and are appropriate, i.e., are not just people’s relatives, spouses.	

*EpiCollect* application has been extensively used in citizen science projects to support user-friendly data collection in poor-resource settings [31, 32]. It also supports managing project data collectors and participants, and, viewing, and retrieval of data. This application is comparable to ‘Our Voice’ Discovery Tool developed by the Global Citizen Science for Health Equity Research Initiative, Stanford University, USA [18]. The EpiCollect 5 (https://five.epicollect.net) was chosen by the project team for data collection, as a secure open-sourced application considered as an easy-to-customise and easy-to-use mobile application suitable for use in low-income settings.

The EpiCollect 5 app is designed by the Imperial College London, and the Big Data Institute at Oxford University (https://www.bdi.ox.ac.uk) and hosted on the world-class cloud hosting provider, Digital Ocean (https://www.digitalocean.com). It is fully GDPR (General Data Protection Regulation) compliant. Data collected will be a secured repository in each country, and only accessed by the designated project team staff. The data will be retrieved and analysed by each country for country-level advocacy purposes, and later on data will be jointly analysed for all countries.

#### Citizen science data extraction and analysis

Once the citizen scientists have completed data collection they will participate in a one-day data extraction/analysis meeting to facilitate their working together to learn, review and interpret the data collected, and refining the methods of exploring CVD risk perception. Data extraction and quick analysis will follow the steps presented in Table 3. Similar to evidence-based participatory citizen science methods, such as *Our Voice,* from which the overall study methods were adapted [33, 34], citizen scientists will work together to agree on interpretation and communication of findings with the assistance of the qualitative researcher(s).

**Table 3:**
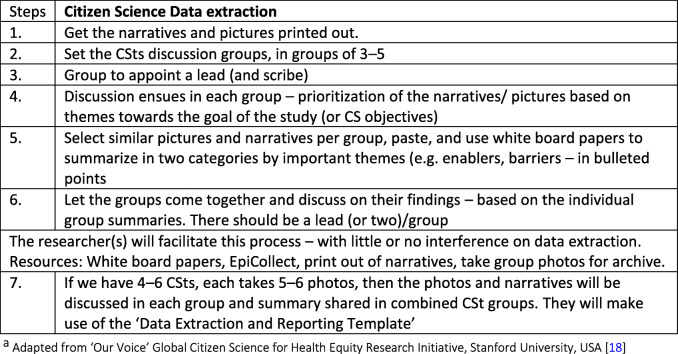
Data extraction and preliminary analysis steps^a^

The first specific objective of this session is to conduct simple analysis of the findings of the pilot data collection (fact-finding). Simple analysis methods that will be adopted include discussing individual narratives and the pictures in groups of 3–5 citizen scientists, summarizing priority issues, and documenting these using cardboard and flip-charts. The second objective of this session is the sharing of the findings of the FGDs (in a summarized format) with the citizen scientists by the qualitative researchers in order for them to identify and agree upon the common themes concerning the concept and perceptions of CVD risk and effective communication of risk. The data analysis meeting will also be used as an avenue for the groups to identify key stakeholders (or persons) that need to be invited to advocacy workshop, including persons that have the potential to influence policy and service implementation in the community. The overall findings from this workshop will be prepared, summarized and made ready for presentation by designated citizen scientists during the advocacy workshop. This will process will be supported by the researchers.

In addition to the simple qualitative data analysis by citizen scientists, the project team will conduct a second level of analysis of the data collected in all the study countries. This will involve recoding and harmonisation of all data collected from the EpiCollect databases in each rural and urban communities in the countries. Data harmonization will also involve downloading the EpiCollect data and carefully recoding the narratives and pictures into quantitatively analysable data. This is for the purpose of supporting comparative data analysis to compare findings in the countries and the project communities.

#### Feedback meeting

Following the data extraction and analysis meeting, a one-day feedback meeting will be held during which designated citizen scientists and the researchers share their observations, experiences, and key findings with a larger stakeholder audience. The aim of this meeting is to facilitate feedback to the community, working together, learning and refining the methods of exploring CVD risk perception, interpretation and communication with the involvement of health caregivers– which is considered crucial for evidence-based care in the community.

### Community advocacy workshop

A one-day advocacy workshop with stakeholders will be held in each study site (rural and urban separately) following data extraction and analysis meetings. The workshop will serve as a time to share the findings of the Citizen Science data collected with a larger set of stakeholders in the communities, and to rehearse the resolutions, key action points and strategies outlined during the feedback session. This will provide a smooth link to learning and using the FGD findings and citizen science data to inform CVD risk screening. Stakeholders to be invited include health workers, CHWs, traditional leaders, church/religious groups, NGOs and opinion leaders in the communities.

A chair will be appointed amongst the stakeholders during or prior to the meeting. Summary findings will be prepared by the citizen scientists in order to reach a consensus on how risk can be communicated effectively, and how advocacy for adoption would be facilitated with the key community stakeholders (including health care workers/facilities) based on the summary findings.

The implications of the findings will be discussed in this forum and the community will be asked to make recommendations. This meeting will serve as a further opportunity for data collection, as well as for advocacy at the community level. The main outcome of this workshop will be the decisions and summary agreements on actions-steps and strategies to be adopted to communicate CVD risk (to improve risk awareness, increase knowledge and support informed decision to seek prompt care). The key stakeholders’ (such as NCD Unit of the Ministry of health, among others) through the meeting chair of the workshop, will write down their responsibilities to supporting community initiative towards addressing the issues raised during the workshop.

### Follow up advocacy – for intervention

Community advocacy and stakeholders’ engagement are avenues expected to engender discussions to co-create solutions such as community-level pilot intervention(s) to address the specific risk factors and accelerators of inaccurate CVD risk perceptions in the communities. The essence of planned follow-up advocacy for the research teams and the citizen scientists is to further meet with stakeholders and organizations such as Ministries of Health, Bureau of Health, NCD units, community health committees, Health Advisory Committees, and Community Extension work programmes to discuss concrete strategies and plans about what kind of interventions the community want to see happen. These strategies could include deployment of CHWs in the communities, households and health-posts (see example in Appendix 1) to conduct community-based campaigns to create awareness around CVD risk, and to take blood pressure and assess raise blood pressure levels. Other expected pilot interventions could include screening at community centre and referral of persons at risk, and training of more counsellors including CHWs, HEWs and Women Development Army (in Ethiopia for example) to support health promotion in the communities. The goal will be to engage with them one-on-one to garner commitment to support pilot interventions prioritized by the citizen scientist during advocacy meetings.

### Data analysis processes

#### Focus group discussions (FGDs)

Prior to the qualitative data analysis, at each study site, the audio-recorded FGDs will be transcribed verbatim in the local languages (Kinyarwanda in Rwanda, Chichewa and Tumbuka in Malawi, and Afan Oromo and Amharic in Ethiopia), and then translated into English. Two experienced qualitative researchers will collaboratively analyse and produce summary findings from the FGDs within 5–6 weeks to enable timely use of findings to support training of the citizen scientist for data collection, presentation and advocacy. Findings will be summarized based on the themes emerging themes during FGD. In order to ensure rigour, the two researchers will collaboratively develop a codebook of themes corresponding after reading the transcripts [35]. Transcripts will be reviewed and compared to check for consistency and additional primary codes and themes. We will use the six steps suggested by Braun and Clarke 2006. These are familiarizing self with the qualitative data; generating initial codes; searching for themes; reviewing themes; defining and naming themes; and producing the report. The narrative summaries of main findings for each theme will be provided and the recurrent themes from FGD participants will be presented in the form of quotes. During feedback session, findings (summaries) of the FGDs will be shared with FGD participants in selected communities for the purpose of validation.

#### Pilot ethnography data extraction and discussion

A simple analysis method will be adopted in line with Citizen science methods of interrogating data collected. This will involve the presentation and discussion on data collected on a daily basis. The community members (i.e. who would later be trained as citizen scientists) will sit together in groups around a table with the research team members, and give feedback of their experience each day. They will also go through the printed pictures and listening to the narratives, to identifying key issues generated during the interview. They will then discuss the issues and context around them, to get an understanding about how people in the community perceive and interpret risk, and would like CVD risk to be communicated to them. This is to give a summary about what maybe expected during a citizen science interview proper.

#### Citizen science interviews

After the citizen science interviews, 8–10 pictures and audio narratives from each citizen scientists at each study site will be generated. The qualitative researchers will facilitate the process of assembling the pictures and narratives, arranging them in themes (based on similarity in the narratives and pictures), and support summarizing, interpretation (or analyse) and discuss. The template developed by ‘Our Voice’ Citizen Science of Stanford University (shown on Table 4) to guide the conduct and recording of data extraction, assembling, and reporting on the key issues identified will be provided by the research team for use [18]. The citizen scientists will identify 2–3 key barriers and or enablers, and paste them on the wall, and discuss the issues and context around them. Also, a list of possible recommendations to support community advocacy will also be generated. The researcher will endeavour not to interfere in the citizen scientists’ analysis of the data.

**Table 4:**
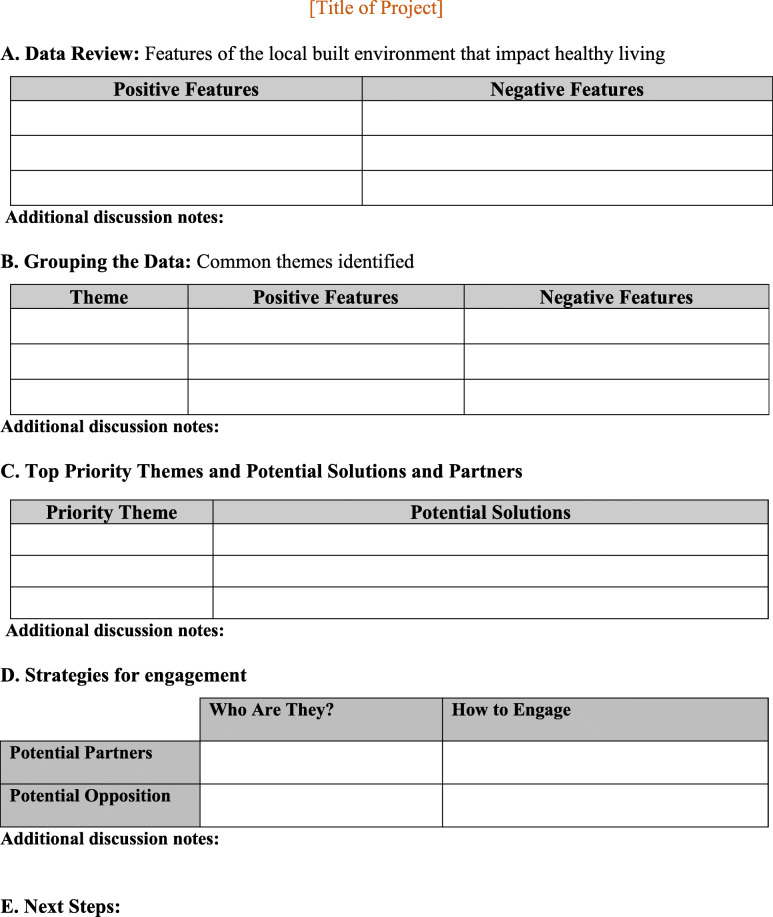
Data extraction and reporting templates

In order to enable joint data analysis and comparison of findings from the rural and urban settings, the data from EpiCollect will be downloaded and captured in a spreadsheet template and harnessed into an analysable form for all the countries. Data analysis will be done using parametric comparisons to further determine the difference in the risk perceptions and its effect on planned health-seeking actions by setting.

### Quantitative data harmonisation and analysis

The recoded participant’s data from the EpiCollect database in all project communities will be harmonised, and pooled for joint analysis, for the purpose of providing a comparison of findings by countries and locations. The participant’s data in the database comprises of demographic variables (viz. country, location, age, and gender), recoded variables (and pictures) on general health risk, perceived CVD risk, perceived threat, communication/presentation of CVD risk, and health seeking behaviour. Descriptive analysis will be undertaken, and this will be followed by inferential analysis, to describe and compared risk perception, presentation, and communication in the project sites. T-test, and ANOVA comparison tests will be undertaken to determine the relationship between risk perception and perceived threat, and presentation of risk among participants in the rural and urban communities. We will also present the geo-spatial map showing participants CVD risk and communication attributes in the countries.

### Research collaborative for implementation

This study, under the CEBHA+ project is facilitated by the Chronic Disease Initiative for Africa (CDIA) in the Department of Medicine, University of Cape Town. We have collaborated with the following research institutions within and outside the CEBHA+ consortium, to support project design and implementation from inception:

- University of Rwanda, University of Malawi; and Malawi Epidemiology and Intervention Research Unit (MEIRU),
- Armauer Hansen Research Institute (AHRI), Addis Ababa, Ethiopia
- Ministry of Health Rwanda; and Rwandan Biomedical Centre
- Research Centre for Health through Physical Activity, Lifestyle and Sport, Division of Exercise Science and Sports Medicine (ESSM), Department of Human Biology, University of Cape Town Faculty of Health Sciences
- Stanford University, for support in Citizen Science implementation, and for providing capacity building for KJO and EVL on their award winning Discovery Tool for ‘Our Voice’ Initiative.

### Activity time line

The implementation for this phase of the CEBHA+ project was planned to begin in July 2019, and end in August 2020. However, due to the unforeseen delays due to logistics, and the current COVID-19 restrictions, the timelines have been moved further. The project implementation is expected to be completed in October 2021 in the four countries, pending the level of lockdown restrictions in the study countries.

## Discussion

This proposed study is part of a larger project that aims to contribute to the development of evidence-informed policies and practices on screening approaches for hypertension, diabetes, and CVD risk in SSA. It is envisaged that using PAR and engaging citizen scientists will more effectively elicit lay understanding and interpretation of the concept of health risk, contributing to the production of more ‘socially robust scientific knowledge’ around this question. In particular, knowledge that is understandable, acceptable and trusted by the community and will assist in the development of relevant, more meaningful risk communication strategies. In this light Asingiswe et al., 2018 previously postulated an integrated conceptual framework to explore how participation in citizen science could be achieved for malaria prevention in Rwanda [36]. This framework stipulated the connective actions (sharing and exchange of malaria-related information), consistent use of malaria preventive and control measures, and collective action for malaria prevention.

There are a number of potential benefits for the citizen scientists and the communities in general, in this project. These are likely to include improved access to scientific information; increased health literacy; a greater understanding of scientific methods and processes and the acquisition of new skills and abilities.

The citizen scientists will certainly gain transferable scientific skills. These will range from skills to conduct community-based survey (qualitative), interviewing, learning data extraction, and simple analysis (putting together the findings), and presentation of findings, and advocacy workshop support.

The strong PAR component of this project gives the citizen scientists the primary role in presenting the data to the community and initiating action to address the problem of CVD. The study also has the potential to yield community-wide benefits such as changes in social values and norms around CVD risk behaviours and building community capacity to access and engage with healthcare workers and policy makers in developing local, contextually appropriate CVD prevention strategies and screening programmes in the four selected countries. Invariably, all stakeholders (including health worker, healthcare managers, community members, and citizen scientists) who participated in implementation of the community-based citizen science projects would have had personal satisfactions and fulfilment as ‘local’ scientists capable of engaging in indigenous science.

### Study limitation and strengths

This study is expected to have some limitations and challenges. It is being undertaken in four CEBHA+ project countries, and the findings are likely to be relevant to and generalisable in countries in SSA with similar socio-economic and cultural environments. There are also expected challenges in the implementation of the study. For instance, in the context of COVID-19, the already agreed activities by the CEBHA+ research implementing team in 2019/2020 (such as community consultations, and community advocacy meetings) will need to be adjusted to accommodate the social distancing regulations that prohibit in-person group meetings. However, the project coordinating team is developing some contingency plan to support virtual, and in-person implementation strategies to address COVID-19 restrictions in the countries.

In addition, the lessons learnt and experience gained through participatory processes will facilitate indigenous knowledge generation, that would support effective population-based CVD risk screening and referrals for care in rural and urban SSA settings. Presumably, a measure of effective communication of risk in the communities, would be the proportion of the “at risk” persons who presented themselves to clinics for early treatment after community-based screening CVD risk and referrals by CHWs. Overall, data obtained will be analysed and findings disseminated using local and international peer review journals, and stakeholders’ forums.

## Conclusion

This study will build capacity at all levels of planned engagement in the conduct of citizen science and participatory research, targeting CVD risk perceptions and collaborative prevention advocacy in SSA. Contextual processes applied and lessons learnt in specific settings (rural and urban) will enable effective redefining or diminishment of boundaries in participatory science to realise effective implementation of sustainable prevention programmes in LMICs.

## Supplementary Information

**Additional file 1.** Ethiopia Administrative and Public Health System overview (Example of country administrative structure and public health system)

## Data Availability

Data sharing is not applicable to this article as no datasets were generated or analysed during the current study.

## Abbreviations

CVD: Cardiovascular disease
SSA: Sub-Saharan Africa
LMICs: Low- and Middle-income countries
CHWs: Community health workers
PAR: participatory action research
CEBHA+: Collaboration for Evidence-based Health Care and Public Health in Africa
NCDs: Non-communicable diseases
SES: Socio-economic status
HEWs: Health Extension workers
FGD: Focus group discussion
CSt: Citizen scientists
CDIA: Chronic Disease Initiative for Africa

## References

[1] Yusuf S, Rangarajan S, Teo K, Islam S, Li W, Liu L (2014). Cardiovascular risk and events in 17 low-, middle-, and high-income countries. N Engl J Med.

[2] Keates AK, Mocumbi AO, Ntsekhe M, Sliwa K, Stewart S (2017). Cardiovascular disease in Africa: Epidemiological profile and challenges. Nature Reviews Cardiology.

[3] Cooney MT, Cooney HC, Dudina A, Graham IM (2011). Total cardiovascular disease risk assessment: a review. Curr Opin Cardiol [Internet].

[4] Lim SS, Gaziano TA, Gakidou E, Reddy KS, Farzadfar F, Lozano R (2007). Prevention of cardiovascular disease in high-risk individuals in low-income and middle-income countries: health effects and costs. Lancet..

[5] Boateng D, Wekesah F, Browne JL, Agyemang C, Agyei-Baffour P, De-Graft Aikins A (2017). Knowledge and awareness of and perception towards cardiovascular disease risk in sub-Saharan Africa: a systematic review. PLoS One.

[6] Surka S, Edirippulige S, Steyn K, Gaziano T, Puoane T, Levitt N (2014). Evaluating the use of Mobile phone technology to enhance cardiovascular disease screening by community health workers. Int J Med Inform [Internet].

[7] Gaziano TA, Abrahams-Gessel S, Denman CA, Montano CM, Khanam M, Puoane T (2015). An assessment of community health workers’ ability to screen for cardiovascular disease risk with a simple, non-invasive risk assessment instrument in Bangladesh, Guatemala, Mexico, and South Africa: an observational study. Lancet Glob Heal.

[8] Abrahams-Gessel S, Denman CA, Montano CM, Gaziano TA, Levitt N, Rivera-Andrade A (2015). The training and fieldwork experiences of community health workers conducting population-based, noninvasive screening for CVD in LMIC. Glob Heart.

[9] Levitt NS, Puoane T, Denman CA, Abrahams-gessel S, Surka S, Mendoza C (2015). Referral outcomes of individuals identified at high risk of cardiovascular disease by community health workers in Bangladesh, Guatemala, Mexico, and South Africa. Glob Health Action.

[10] Surka S, Steyn K, Everett-Murphy K, Gaziano TA, Levitt N. Knowledge and perceptions of risk for cardiovascular disease: Findings of a qualitative investigation from a low-income peri-urban community in the Western Cape, South Africa. African J Prim Heal Care Fam Med. 2015;7(1). http://phcfm.org/index.php/phcfm/article/view/891. 10.4102/phcfm.v7i1.891 PMC4656922 26842511

[11] Okop KJ, Mukumbang FC, Mathole T, Levitt N, Puoane T (2016). Perceptions of body size, obesity threat and the willingness to lose weight among black south African adults: a qualitative study. BMC Public Health.

[12] Gigerenzer G, Gaissmaier W, Kurz-milcke E, Schwartz LM, Gigerenzer G, Gaissmaier W (2007). Helping doctors and patients make sense of health statistics. Assoc Psychol Sci.

[13] Okop KJ. Exploring the association between body image, body fat, and total cardiovascular disease risk among adults in a rural and an urban community of South Africa. University of the Western Cape. https://etd.uwc.ac.za/handle/11394/5599 (2017).

[14] Bovet P, Chiolero A, Paccaud F, Banatvala N. Screening for cardiovascular disease risk and subsequent management in low and middle income countries: Challenges and opportunities. Vol. 36, Public Health Reviews. 2015. 10.1186/s40985-015-0013-0 PMC5804497 29450041

[15] Payne YA (2017). Participatory action research. The Wiley-Blackwell Encyclopedia of Social Theory.

[16] Morton Ninomiya M, George N, George J, Linklater R, Bull J, Plain S (2020). A community-driven and evidence-based approach to developing mental wellness strategies in first nations: a program protocol. Res Involv Engagem.

[17] Abayneh S, Lempp H, Hanlon C, Branquinho C, Tomé G, Grothausen T (2020). Community-based youth participatory action research studies with a focus on youth health and well-being: a systematic review. J Community Psychol.

[18] King AC, Winter SJ, Chrisinger BW, Hua J, Banchoff AW (2019). Maximizing the promise of citizen science to advance health and prevent disease. Prev Med (Baltim).

[19] Den Broeder L, Devilee J, Van Oers H, Schuit AJ, Wagemakers A (2018). Citizen science for public health. Health Promot Int.

[20] McKinley DC, Miller-Rushing AJ, Ballard HL, Bonney R, Brown H, Cook-Patton SC (2017). Citizen science can improve conservation science, natural resource management, and environmental protection. Biol Conserv.

[21] Barrie H, Soebarto V, Lange J, Corry-breen FM, Walker L, Katapally TR (2019). The rise of citizen science in health and biomedical research. Am J Bioeth [Internet].

[22] Dickinson JL, Shirk J, Bonter D, Bonney R, Crain RL, Martin J (2012). The current state of citizen science as a tool for ecological research and public engagement. Front Ecol Environ.

[23] Becker A (1997). Book reviews : Alan Irwin: citizen science. A study of people, expertise and sustainable development. In: organization studies.

[24] Wallerstein N, Duran B, Oetzel JG, Minkler M. Community-based participatory research for health: Advancing social and health equity: Wiley; 2017. p. 19–29.

[25] Rehfuess EA, Durão S, Kyamanywa P, Meerpohl JJ, Young T, Rohwer A (2016). An approach for setting evidence-based and stakeholder-informed research priorities in low- and middle-income countries. Bull World Health Organ.

[26] Macdonald CD (2012). Understanding participatory action research: a qualitative research methodology option. Can J Action Res.

[27] Vollman AR, Anderson ET, McFarlane J. Canadian community as partner: Theory and multidisciplinary practice. First. (ESP). WKHA, editor. Canadian Community As Partner: Theory and Multidisciplinary Practice. 2016. 1–908 p.

[28] Kemmis S, Wilkinson M. Participatory action research and the study of practice. In: (Eds.) D& YSL, editor. Action research in practice; partnership for social justice in education. 1998. p. 336–96.

[29] Beauchemin C, González-Ferrier A (2011). Sampling international migrants with origin-based snowballing method. Demogr Res.

[30] Hinckson E, Schneider M, Winter SJ, Stone E, Puhan M, Stathi A, et al. Citizen science applied to building healthier community environments: advancing the field through shared construct and measurement development. Int J Behav Nutr Phys Act [Internet]. 2017;14(1):133. http://ijbnpa.biomedcentral.com/articles/10.1186/s12966-017-0588-6. 10.1186/s12966-017-0588-6 PMC5622546 28962580

[31] Heigl F, Zaller JG (2014). Using a citizen science approach in higher education: a case study reporting Roadkills in Austria. Hum Comput.

[32] Doane J, Schoenhals S, Sherpa A, Lama L, Fassl B, Levy D (2019). Implementing a mobile app for field global health research: successes, challenges, and future directions. J Investig Med.

[33] Odunitan-Wayas FA, Hamann N, Sinyanya NA, King AC, Banchoff A, Winter SJ, et al. A citizen science approach to determine perceived barriers and promoters of physical activity in a low-income south African community. Glob Public Health [Internet] 2020;0(0):1–14. 10.1080/17441692.2020.1712449. 10.1080/17441692.2020.1712449 31992139

[34] King AC, King DK, Banchoff A, Solomonov S, Ben NO, Hua J (2020). Employing participatory citizen science methods to promote age-friendly environments worldwide. Int J Environ Res Public Health.

[35] Braun V, Clarke V (2006). Using thematic analysis in psychology. Qual Res Psychol.

[36] Asingizwe D, Poortvliet PM, Koenraadt CJM, Van Vliet AJH, Murindahabi MM, Ingabire C (2018). Applying citizen science for malaria prevention in Rwanda: an integrated conceptual framework. NJAS - Wageningen J Life Sci.

