# Supplementary figures and images for: Community-driven citizen science approach to explore cardiovascular disease risk perception, and develop prevention advocacy strategies in sub-Saharan Africa: a programme protocol
Source: Res Involv Engagem. 2021 Feb 26;7:11. doi: 10.1186/s40900-020-00246-x (PMC7907793; doi:10.1186/s40900-020-00246-x)

**Supporting Information**: Ethiopia Administrative and Public Health System overview


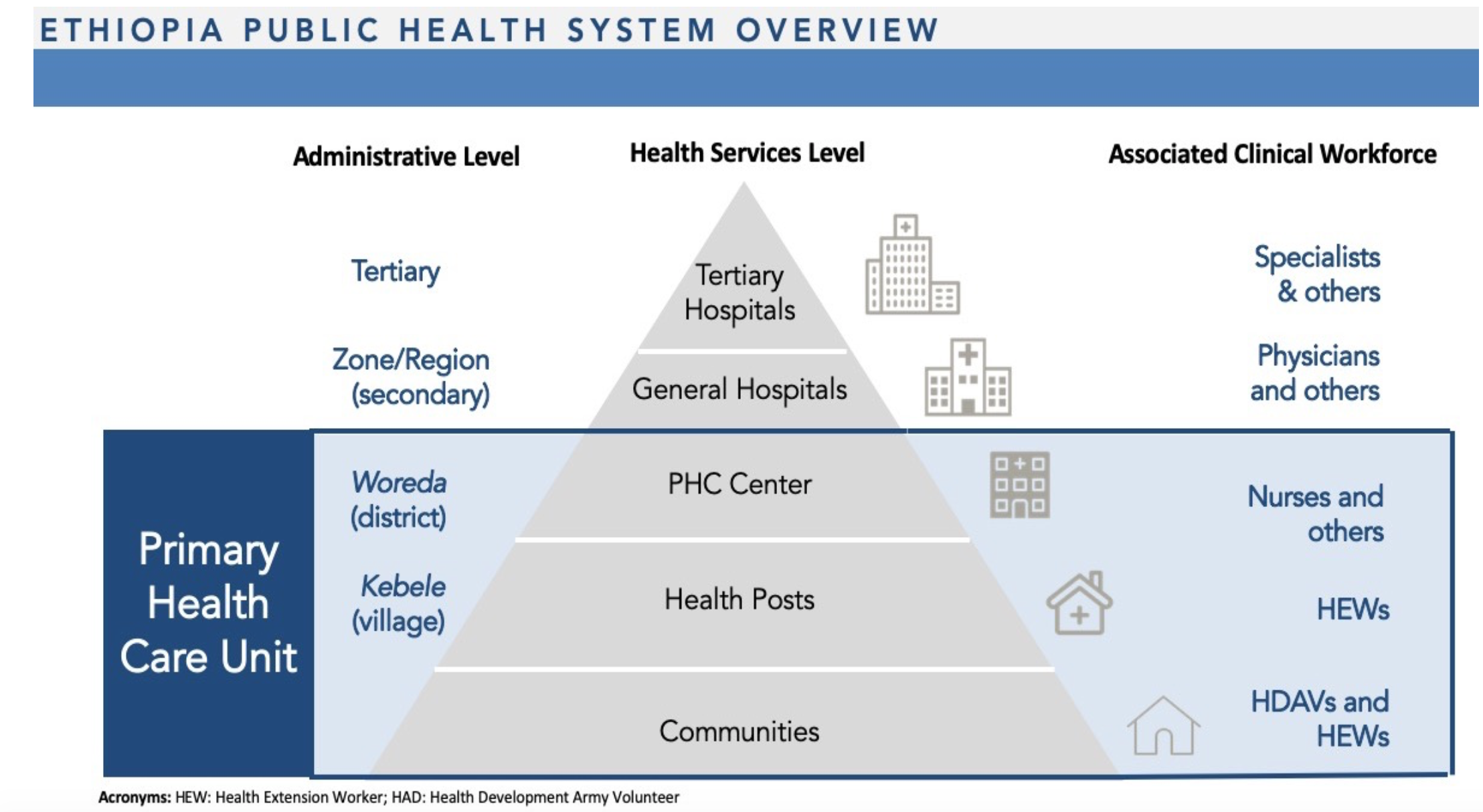

Supplement: Supplementary file 1 — Additional file 1. Ethiopia Administrative and Public Health System overview (Example of country administrative structure and public health system) [file 40900_2020_246_MOESM1_ESM.docx]
